# Empowerment of Youth With Intellectual Disabilities Through Inclusive Research in Africa

**DOI:** 10.1111/jar.70301

**Published:** 2026-08-28

**Authors:** Chioma Ohajunwa, Anthea Hansen, Lieketseng Ned, Theresa Lorenzo

**Affiliations:** ^1^ Africa Centre for Inclusive Health Management, Faculty of Economic and Management Sciences Stellenbosch University Stellenbosch South Africa; ^2^ Department of Health Professions Education, Faculty of Medicine and Health Sciences Stellenbosch University Tygerberg South Africa; ^3^ Division of Disability and Rehabilitation Studies, Department of Global Health, Faculty of Medicine and Health Sciences Stellenbosch University Stellenbosch South Africa; ^4^ Division of Disability Studies, Department of Health and Rehabilitation Sciences, Faculty of Health Sciences University of Cape Town Cape Town South Africa

**Keywords:** Africa, empowerment, inclusive research, intellectual disability, social justice, youth development

## Abstract

**Background:**

Despite growing global interest in participatory research, the practice of including persons with intellectual disabilities as co‐researchers remains largely underexplored within the African context. This paper reflects on the methodological processes of implementing inclusive research with youth with intellectual disabilities, including the lessons learned during this process.

**Methods:**

Youth with intellectual disabilities were recruited and trained as co‐researchers to conduct interviews as part of a pilot study for the qualitative evaluation of the Special Olympics Healthy Communities Programme in South Africa. The core team consisted of four co‐researchers and three university researchers.

**Results and Conclusions:**

The reflections and lessons learned from the research team demonstrated that conducting inclusive research with youth with intellectual disabilities is beneficial to all involved. It is achievable and speaks to the reciprocity of equity, inclusion, empowerment and social justice.

## Summary of Key Outcomes

- There is a need for increased praxis regarding inclusive research with persons with disabilities in Africa.
- Persons with intellectual disabilities bring a wealth of information from lived experience to inform inclusive research collaboration and outcomes.
- Including persons with intellectual disabilities in research will showcase their capabilities and help change prevalent stigma and marginalisation of persons with disabilities and their families.
- This paper demonstrates that inclusive research does not have to be resource‐intensive for researchers, as experiences of persons with intellectual disabilities contribute to a rich human resource.

## 1 Introduction

Persons with intellectual disabilities have been marginalised in the field of research (Kahonde 2023; O'Brien et al. 2014). Society's deficit view and exclusion of youth with intellectual disabilities in research and beyond disempower them (Adnams 2018). Despite growing global interest in participatory research, the practice of including persons with intellectual disabilities as co‐researchers remains largely underexplored within the African context (Adnams 2018). This paper reflects on the methodological processes of implementing inclusive research with youth with intellectual disabilities and the lessons learned during this process. Longstanding calls for inclusive research have been advocated for through various international legislation such as the United Nations Convention on the Rights of Persons with Disabilities (CRPD) (UN 2006), which calls for inclusion across all aspects of life as a right.

Inclusive research with persons with intellectual disabilities is increasing globally (Hewitt et al. 2023). A systematic review of inclusive research with persons with intellectual disabilities, conducted from the year 2000, revealed that inclusive research is occurring across various contexts in Europe, the United States, the United Kingdom, Australia and other Western countries, spanning issues of sexual health; physical and mental health, including parenting; amongst other topics (Hewitt et al. 2023; Walmsley et al. 2018). However, there are fewer inclusive research initiatives occurring in resource‐constrained or so‐called developing countries, such as Africa, the context where we are based (Kahonde 2023; O'Brien et al. 2014; Wanjagua et al. 2024).

Inclusive research is not only about the inclusion of persons with intellectual disabilities but spans persons with any disability and includes a range of methods, models and practices of implementing research. It is worth noting that there are various understandings of the terminologies used within inclusive research, some of which are contested (Walmsley et al. 2018). The use of terms such as inclusive research, co‐researcher, participant researcher and others for persons with intellectual disabilities within research collaborations should be considered within the context in which the research unfolds (Walmsley et al. 2018; Strnadová and Walmsley 2018). Historically marginalised and excluded from health and social research, Walmsley (2001) coined the term ‘co‐researcher’ to connote a more equal partnership within the research process. It is also referred to as collaborative, emancipatory, action or participatory research (Garcia‐Lee et al. 2024; Hart 2024), even though, as Walmsley et al. (2018) note, the strengths of the researchers and their contributions may differ. Although the terminology ‘co‐researcher’ may not be deemed to be equitable to other researchers (Gjermestad et al. 2023), for this paper we align with the collaborative and emancipatory perspective of the term ‘co‐researchers’ offered by Walmsley et al. (2018).

The skilling and training of youth with intellectual disabilities as co‐researchers encourages inclusive research practices. Two decades of inclusive research have shown that it may demand more time and resources, human and financial, than regular research (Walmsley et al. 2018), and more effort and negotiations with ethics committees to ensure no harm while maintaining research rigour (Kahonde 2023). While scholars have continued to advocate for inclusive research, there remains an assumed cognitive incompetence of persons with intellectual disabilities that is cited as a reason for exclusion (Wanjagua et al. 2024; Capri and Coetzee 2012). This exclusion often occurs more frequently within some contexts, where certain pervasive cultural and religious belief systems, financial difficulties, and structural challenges can influence perceptions of intellectual disabilities (Mkabile et al. 2021). This results in a denial of opportunities for persons with intellectual disabilities to contribute their unique insights to research. These unique insights need to be prioritised.

We argue that an inclusive research approach may also be seen as a matter of social justice (Hart 2024) as it contributes to the empowerment and emancipation of these youth, affirming their capacity to equally contribute to society. Importantly, working with and including youth with intellectual disabilities is also beneficial to the academics within the research team (McConkey et al. 2018).

Researchers are called to adopt approaches that are emancipatory and support the inclusion and full participation of persons with disabilities (O'Brien et al. 2014; Embregts et al. 2018). Both the United Nations CRPD (UN 2006) and the World Report on Disabilities (WHO 2011) highlight the importance of understanding lived experiences and recognising the valuable contributions made by persons with disabilities. The World Report on Disabilities (WHO 2011) highlights that many barriers continue to affect the full and equal participation of persons with disabilities. The International Association for the Scientific Study of Intellectual and Developmental Disabilities (IASSIDD) Special Interest Research Group (SIRG) has argued that there is minimal inclusive research, despite its potential to facilitate a reduction of some of these barriers (Kahonde 2023). There is insufficient literature from African intellectual disability researchers on inclusive research global platforms (Wanjagua et al. 2024), and a call was made for more African voices to contribute to the global discourse on inclusive research (Kahonde 2023; Wickenden 2024). This paper is aimed at fulfilling this mandate by contributing a South African experience to the global discourse of inclusive research.

## 2 Literature Review: Intellectual Disability, Health and the African Context

The World Health Organization (2022) reports that approximately 200 million people have significant disabilities, including persons with intellectual disabilities. It is estimated that 1%–2% of the world's population has an intellectual disability, with most of these persons living in low‐ and middle‐income countries (LMICs) (Maulik et al. 2011; Kleintjes et al. 2020). In 2013, the global burden of disease report estimated that there were 154 million people with some level of intellectual disability (Vos et al. 2015). Researchers note that the prevalence of intellectual disability is likely to be higher and more debilitating in Africa owing to the effects of poverty and poor nutrition as well as inadequate and inappropriate services (Adnams 2018). Furthermore, the disempowerment of youth with intellectual disabilities is still more prevalent within contexts shaped by structural and resource constraints compared to settings with greater access to resources and support (Kahonde 2023). Within the African context, people with intellectual disabilities are found to be amongst the poorest, most vulnerable and marginalised groups, and they display significant health needs (Cooper et al. 2004; Kleintjes et al. 2020).

In many African countries, disability remains traditionally stigmatised (Njenga 2009; Baffoe 2013; Koszela 2013). There are legislative frameworks and policies across Africa that seek to uphold the rights of persons with disabilities, including intellectual disability (WHO 2011) and to mobilise resources to actively address systems and structures that perpetuate the disempowerment of these individuals. For example, many African countries are signatories to the CRPD and the United Nations Convention on the Rights of the Child (UNICEF 1989). Additionally, in South Africa, frameworks such as the South African National Department of Health Mental Health Policy Framework and Strategic Plan (South Africa National Department of Health 2013) and the National Mental Healthcare Act (South African Government 2002) address the rights of mental healthcare users, including users with intellectual disability (Adnams 2010). Despite these progressive legislative frameworks, few inclusive policies have been effectively implemented for the benefit of persons with intellectual disabilities.

### 2.1 Exploring Inclusive Research

Inclusive research has been noted to be an evolving concept (O'Brien et al. 2014; Seale et al. 2014; Nind 2019; Walmsley et al. 2018) that includes a variety of methods or approaches to research that are participatory, emancipatory and action‐based. Integral to these approaches is the principle of moving away from only including people as subjects of anthropological and historical research to allowing meaningful involvement across the levels of the research process from conceptualisation, research design, data collection, analysis and dissemination (Nind and Vinha 2014; Bigby et al. 2014), allowing diverse identities, experiences and narratives to influence research outcomes (Baldwin‐White 2024). Inclusive research not only speaks to persons with disabilities across the age span and experiences (Kahonde 2023) but also to community members and various vulnerable and marginalised populations (Sikapa et al. 2024). However, issues of politics of voice, representation, and identity remain a concern. Inclusive research facilitates positive identity for youth with disabilities (Kuhlmann et al. 2024) and all persons with disabilities. Internationally, there has been a growth in the inclusion of persons with intellectual disabilities in research (Kyriazis et al. 2023; Strnadová and Walmsley 2018) through various roles such as paid research assistants, co‐facilitators and advisory team members (O'Brien et al. 2014). However, in an LMIC, the situation is one of limited inclusive research.

Inclusive research in Africa has been fraught with many challenges, which include the medicalisation of disability, where the focus is more on providing medical care to persons with intellectual disabilities while neglecting their social needs (Wanjagua et al. 2024; Walmsley et al. 2018; Kahonde 2023). Factors such as gatekeeping by parents and caregivers, community and self‐stigma, safety and mistrust, and a sense of powerlessness by caregivers present as barriers to research. Despite these challenges, engaging in inclusive research will contribute to the emancipation of youth with intellectual disabilities (Mkabile et al. 2021). We align our voices with this argument and posit that inclusion should be the norm and not the exception, benefiting persons with intellectual disabilities, university academic researchers and policymakers (McConkey 2018).

Many studies in sub‐Saharan Africa have explored various aspects of the lived experiences of persons with intellectual and developmental disabilities, for example, Ghana, Nigeria, Cameroon, Sierra Leone and Kenya. These studies were focused on the creation of more inclusive societal spaces (Hervie 2023; Gyimah et al. 2024); inclusive education and service provision (Okyere et al. 2019; Mills 2019; Mwangi et al. 2023); cultural and religious attitudes (Ackah‐Jnr and Appiah 2025; Miezah et al. 2025; Opoku et al. 2021); the use of digital resources for learning, access to justice and advocacy (Akin‐Fakorede et al. 2025; Arimoro 2019; McKenzie and Ohajunwa 2017); and difficulties of navigating ethics committees and the need for using accessible language as drivers of inclusive research (Wanjagua et al. 2024). However, there is inadequate mention of studies that include persons with intellectual and developmental disabilities as co‐researchers in the study. This paper speaks to that gap, ensuring that inclusive research within the African context not only yields relevant contextual and indigenous knowledge but also supports social change (Sikapa et al. 2024).

## 3 Methodology

This paper seeks to contribute a reflection on the lessons learned during the methodological process of educating and training youth with intellectual disabilities in research processes as co‐researchers for a pilot study of the Special Olympics Healthy Communities Evaluation project in South Africa (McConkey 2018). McConkey (2018) has reported on the wider findings of the Special Olympics Healthy Communities Evaluation project. We draw on the action learning cycle (CDRA 2005), adapted from Taylor et al. (1997), to make sense of our experience of doing co‐research. The framework operates in a cyclical manner, moving through four interconnected components—(1) action (experience), (2) reflection, (3) learning and (4) planning.

### 3.1 Step 1. Action—Outlining Our Co‐Research Experience

In the action section, we recount our experiences of engaging in co‐research with persons with intellectual disabilities, guided by prompts such as: where did the experience happen?, what happened?, who was involved? and what did they do?

First, we outline the influencing and overarching programme—Special Olympics International (SOI) Healthy Communities—that aimed to promote the health of youth with intellectual disabilities through education targeted at the individual who participated in their Healthy Athletes Programmes and their family (McConkey 2018). We were involved as researchers in an evaluation process of the SOI Healthy Communities programme. South Africa was the pilot site for the evaluation. Youth with intellectual disabilities were involved as co‐researchers. The main research question of the evaluation was: How do Healthy Community programmes bring about improved health and healthier lifestyles for youth with intellectual disabilities?

#### 3.1.1 The Research Team of the Pilot Study

The team for this pilot study involved three university researchers and four youth with intellectual disabilities (two males and two females) who were trained as co‐researchers. Bigby et al. (2014) posit that there are three main ways of conducting research with persons with intellectual disabilities. (1) The persons with intellectual disabilities act as advisors; or (2) they lead the research process; and/or (3) persons with and without intellectual disabilities work together, taking different jobs based on their capabilities. In this pilot study, we adopted the first and third approach. The co‐researchers acted as advisors and collaborated as part of the research team. The co‐researchers informed the data generation tools, gathered data, disseminated study outcomes, and expressed choice and autonomy by informing the team regarding acceptable ethical considerations for the research. All of the co‐researchers were also beneficiaries of the SOI Healthy Communities programme and could be considered as insider researchers (Berger 2015).

#### 3.1.2 Positioning the University Researchers

As the university researchers, we are deeply committed to fostering inclusive practices; we acknowledge that who we are shapes all our research endeavours. We all have wide‐ranging experience working with persons with disabilities; as clinicians (A.H., L.N. and T.L.), we have worked in clinical and community contexts with persons with disabilities. As educationalists and disability scholars (C.O., A.H., T.L. and L.N.), we have worked across the educational context with persons with disabilities. While none of us has a disability, we have actively pursued advancing the rights of persons with disabilities through our academic scholarship as well as our community‐oriented work for more than a decade. Throughout this project, we challenged our assumptions and beliefs by adopting a critically reflexive approach as well as through dialogue and reflection amongst the researchers and co‐researchers.

#### 3.1.3 Research Paradigm and Approach

Situated within an interpretive paradigm, the pilot study adopted a qualitative participatory methodology (Creswell and Poth 2016). The inclusive design was also aligned to our aim for a critical emancipatory approach (CER) and co‐construction of knowledge with our co‐researchers (Mertens 2009). CER promotes participatory engagement, inviting shared decision‐making and including sectors of society who are otherwise left out. CER is premised on equality and equity, advocates for social justice, peace and team spirit, and seeks to foster changes in people's hearts and minds (Xolisile and Bekithemba 2021). Our study design was implemented in a manner that was participatory and inclusive of youth with intellectual disabilities (Bigby et al. 2014). CER informed the methodological implementation of the pilot study. CER influenced our engagement with our co‐researchers (youth with intellectual disabilities). We constantly encouraged their agency and critical thinking through ongoing discussions with the co‐researchers so that their opinions counted towards a more transformative methodological process for the pilot study (Mokotjo 2024; Mahlomaholo 2009; Nkoane 2012).

#### 3.1.4 Study Context

Mitchell's Plain suburb in the Western Cape Province of South Africa was selected for the pilot study, as it was an area where the SOI Healthy Communities programme was actively operating in, and the researchers knew the community rehabilitation workers who continued to support the co‐researchers afterwards. The co‐researchers, (residents of Mitchell's Plain) were recruited from a school in the Lenteguer area of this suburb. This area is considered a historically disadvantaged area in the Western Cape, still plagued by a lower socio‐economic status and impoverished conditions. Mitchells Plain is mainly an Afrikaans‐ and English‐speaking community. Some social determinant factors include high unemployment, a significant crime rate, partnered with alcohol, drug abuse and gang‐related activities. These factors are linked to the low levels of education and training in this area, as well as the impact of the legacy of apartheid on these disadvantaged communities (City of Cape Town 2022).

#### 3.1.5 Recruitment and Sampling Strategy for Co‐Researchers

The target population for co‐researchers was youth with intellectual disabilities who were SOI participants. We partnered with SOI coordinators in the region to recruit the youth, and the research project was explained to them in person with the support of pictorial information sheets. Nine youth indicated interest in participating as co‐researchers.

Through purposive sampling, six youth were selected to be trained. The inclusion criteria to be a co‐researcher were that they were required to be involved in the activities of the Special Olympics Healthy Athletes and/or the Healthy Communities programme. They had to be able to communicate independently (speak, write or draw to express their thoughts) so that they could intellectually understand the study and give consent. Their capacity to be available in the community school, which was the site for the training and research, was also considered. No additional sociodemographic criteria, such as gender or education, were applied, as the focus was on youth with intellectual disabilities' participation in the SOI Healthy Communities programme and their capacity to contribute meaningfully to the research process. The exclusion criteria applied were youth with intellectual disabilities who were not part of the SOI activities and were unable to communicate independently, and were unable to intellectually understand the study and give consent. The reason for this exclusion criterion was that we sought to engage with the co‐researchers for feedback on the ethics of implementation, on what is acceptable or not, so this intention required their ability to communicate their thoughts independently. Two youth dropped out due to personal circumstances. Four youth with intellectual disabilities were trained as co‐researchers for this pilot study. The age range of the co‐researchers was 18–20 years, with equal representation of two males and two females.

#### 3.1.6 The Workshop Methodology for Training the Co‐Researchers

The co‐researchers' training followed two stages: The first stage involved a 2‐day workshop. The second stage included a follow‐up and recap training session a week later.

##### 3.1.6.1 Workshop Phase 1

A 2‐day workshop was attended by three university researchers and four youth with intellectual disabilities. At the start of the workshop, time was taken to build rapport and get to know the co‐researchers and their interests. The university researchers once again explained the purpose of the research, the project focus and why the evaluation team wanted to conduct inclusive research. We also explained how important the views of the co‐researchers were to the process and sought permission to record the training, which they granted. A simply worded, pictorial consent form, drafted from the university consent form template, was given to the co‐researchers to sign. The university researchers explained the rights of the co‐researchers and responded to all questions and queries before signing the consent forms. Consent was also sought from their parents, as they were a vulnerable group, even though they were legally of age.

We prepared a PowerPoint presentation with an accompanying simplified training manual with pictorial illustrations that was given to the co‐researchers to support their learning and for future reference. Information in the manual was aligned to the PowerPoint presentation to eliminate confusion. The training manual had simplified explanations of what research is and how to have conversations (interviews) with people.

After we worked through the manual, the co‐researchers were paired up and asked to explain their understanding of the research and interview questions. Next, they reviewed the manual and interview schedule again while in pairs and provided some verbal feedback on the interview questions. The university researchers sat with each pair of co‐researchers intermittently and joined their conversation to facilitate more discussion when needed.

The co‐researchers were trained on how to conduct interviews, which involved practical role‐play of interviews. With the permission of the co‐researchers, the training sessions were audio‐recorded, and after each practical role‐play of the interviews, we asked the co‐researchers to give us verbal feedback. The university researchers who facilitated the training also documented their experiences in their reflective journals on the training activities. Based on the feedback from the co‐researchers given during the 2‐day workshop, the university researchers took a week to consolidate and revise the interview schedules, which had both structured (for demographics) and open‐ended questions.

##### 3.1.6.2 Workshop Phase 2

This phase involved a 1‐day follow‐up workshop to recap the information shared in the first workshop. This workshop was conducted 1 week after the initial workshop. It involved confirming consent with the co‐researchers, reflecting on what co‐researchers learnt, and a role‐play of doing interviews between researchers and co‐researchers using the revised interview schedule.

#### 3.1.7 Data Gathering With Co‐Researchers

Logistical issues were arranged before the interviews by the university researchers. As preparation on the day of the interviews, the research team had checked in with the co‐researchers to explore how they felt. We addressed any concerns or worries that they had and checked whether they still wanted to continue. The co‐researchers were happy to continue with the research.

The co‐researchers conducted in‐depth, face‐to‐face interviews with four athletes (athletes is the term used by SOI for youth with intellectual disabilities who are participants in SOI activities) at a school for skills within the co‐researchers' community. Two co‐researchers were paired with a university researcher, and the interview was conducted together. One co‐researcher led the interview, and the second co‐researcher observed. This process was alternated between participants so that each co‐researcher had an opportunity to lead the interview. Each interview was approximately 20 min long, and participants were asked if they would prefer the interview to be conducted in English or Afrikaans (which are prevalent languages in the study context). All participants chose English.

#### 3.1.8 Data Analysis

The pilot data gathered for the SOI study was translated and transcribed verbatim and thematically analysed by the university researchers following the six‐step process of Braun and Clarke (2006). Although co‐researchers were not involved in this formal data analysis, each interview they collaborated on was discussed with them. In alignment with the CER approach, this allowed for reflexive engagement and collective sense‐making, ensuring the co‐researchers' perspectives were incorporated into the pilot study. This process also contributed to building the confidence and skills of the co‐researchers.

#### 3.1.9 Ethical Considerations

Ethical approval for the SOI pilot study was obtained from the Human Research Ethics Committee of the Faculty of Health Sciences, University of Cape Town (HREC668/2014). Participation was voluntary and informed consent was obtained. Co‐researchers were free to withdraw from the study at any stage. Justice calls for the fair treatment of research participants, free from discrimination, as well as fairness in the distribution of benefits and burdens of the research. To reflect justice, we ensured that the pilot study benefitted persons with intellectual disabilities and their families, enhancing contextual, personal and societal benefits (Schwartz et al. 2020) by informing the development of programmes that further enhance their health and wellbeing. We conducted the interviews within their school as we were cognisant of their safety, thus upholding ethical principles of beneficence and non‐maleficence.

### 3.2 Steps 2 and 3: Reflections and Lessons Learnt on Implementing Inclusive Research

In this reflection and learning section, we critically reflect on the inclusive process of the pilot project, asking why events unfolded as they did, what enabled or constrained meaningful participation and how our own assumptions shaped the process. We draw out the key insights that emerged from this experience, and we include quotations from the debriefing session with the co‐researchers to support the reflections and interpret these reflections in relation to the existing literature on inclusive research.

Working with people with disabilities as active participants in research and increasingly as co‐investigators as part of research teams, is producing more authentic data, giving insights into their worlds and concerns…. (Wickenden 2024, 3)

Wickenden's quote above illustrates the importance of implementing inclusive research, in agreement with our focus here. During the process of this study, the co‐researchers were recognised for their expertise related to living with an intellectual disability. Wickenden (2024) highlighted that the co‐researchers' experiential knowledge related to disability was key in eliciting narratives of being included in sports from their peers who were the research participants.

We frame our reflections according to four themes: (1) Addressing power dynamics and creating a comfortable space; (2) Body language; (3) A safe space for reflecting and (4) Ensuring process consent. These themes offered important considerations to foster an inclusive and collaborative research process.

#### 3.2.1 Theme 1: Addressing Power Dynamics

The first lesson we learnt was to give adequate time to planning. Bailey et al. (2014), in their research on the inclusion of children with disabilities and youth as partners in research, identify time as a critical component of inclusive research. We recognised that we were engaging with vulnerable youth who come from historically marginalised communities. We also noted that everyone has individual identities, that power dynamics exist, and that these factors contribute to building relationships. So we planned for more time to ensure we addressed all power dynamics and helped the youth feel at ease, seeking to conduct inclusive research that was an empowering process for all involved (Hurtubise and Joslin 2023). We were conscious that this opportunity was the first time these youth with intellectual disabilities were invited to be involved in research as co‐researchers. We noted that they carried certain anxieties related to themselves as individuals and related to the research processes. Their anxiety can be seen in an honest claim:

… I'm very nervous. (CR2)

These anxieties were revealed in our initial, informal conversations with the youth. They had anxieties about whether they were ‘qualified’ to do research, whether they would be able to remember what to ask and how to ask it, and anxiety around how their peers and other participants would react to them being co‐researchers. Frankena et al. (2019) advocate that creating a space for shared decision‐making and where everybody's voice is heard is important for inclusive research. We mediated the power dynamics through regular, informal conversations about the family, school and events that occurred in their communities (Wickenden 2024). These were safe, comfortable topics that helped to open up the co‐researchers and build their confidence as part of the research team. Members of the team who spoke their local languages also had conversations with the co‐researchers in their local language during the research process, which was very encouraging to the co‐researchers.

Beyond creating safe psychological spaces, consideration of the feasibility of roles as well as practical aspects is important when planning with the people with intellectual disabilities as co‐researchers (Frankena et al. 2019). These considerations are different for every context. For instance, within the context of South Africa's history of apartheid and the Group Areas Act of 1950, a forced relocation of Black people against their will was implemented, and communities were segregated according to race, resulting in previously disadvantaged communities being placed far from the main city (Roos et al. 2014). Taking this into account, we ensured proximity by conducting the study within the co‐researchers' community as a form of support. We chose their community not only to create comfort and familiarity but to equally address any power dynamics that may exist (Wickenden 2024). Additionally, efforts taken in this research fostered the actualisation of rights of these youth; inclusion of persons with intellectual disabilities within society addresses one of the critical pillars of sustainable development—social sustainability. Social sustainability is aligned with the United Nations Sustainable Development Goals (United Nations 2015), which include the area of research. In this way, inclusive research contributes to social cohesion for communities (Carnemolla et al. 2021).

#### 3.2.2 Theme 2: Body Language

Second, we paid attention to our body language to be sure to communicate not only with words but also to ensure that we were conscious of our actions and how we positioned ourselves, so that the process of getting to know each other was non‐threatening. One of the co‐researchers affirmed the importance of body language in communicating, stating that he also was watching the pilot study participants and us and knew when participants became comfortable. When asked by one of the university researchers about how he knew they were comfortable, he stated:

First, she sat down, then she took off her shoes. Then she sat like this and afterwards she relaxed. (CR1)

This response confirmed the relevance of consciousness to represent appropriate body language. This awareness helped to mediate the power dynamics between university researchers and the co‐researchers that contributed to rapport building. The entire research team would sit on the floor sometimes or sit in a circle, using Gibb's reflective cycle approach to write and talk about our thoughts, feelings and actions related to the research process (Markkanen et al. 2020).

#### 3.2.3 Theme 3: Creating a Safe and Comfortable Space for Reflecting

Third, after each interview, a debriefing session was held, and co‐researchers were asked to reflect on what they enjoyed about the interviews and what they found challenging. Through these reflective spaces, we began to see the evidence of the co‐researchers' confidence growth as they engaged with the university researchers and peer participants. Co‐researchers began to discuss their experiences more openly and expressed appreciation for the training and support they received during the interview process, noting that they would like to be involved in future research as well. The co‐researchers were acutely aware of themselves in the process and were accurate in their self‐analysis. A co‐researcher reflects this appreciation:

The difference was that you were there to help me through the interview and help me explain the questions to the person if they didn't understand. Then you were there to help me… at first I couldn't talk to people like that, look at them in their face, in their eyes. I couldn't talk to people like that but now I can. (CR1)

The need for skills acquisition and capacity building was a key reason for training and partnering with persons with intellectual disabilities in research. We were glad to see this aim achieved in an extract from the feedback of a co‐researcher:

I learned a lot being the interviewer, how to approach the person and how to talk to the person, and also to listen to the person, what he's saying. You mustn't interrupt until the person has finished what they're saying. (CR2)

The co‐researchers were committed to the training and the research process. They were responsive to what the participants were saying. For example, one co‐researcher would respond, ‘that's nice’ to an answer, and the participant would nod and smile. This affirmation created rapport with the research participant. In creating a comfortable space with the co‐researchers, we inadvertently also influenced their engagement with the pilot study participants.

As researchers, we experienced that in collaborating in research with persons with intellectual disabilities, the focus should not only be on the preparation of accessible content, but also on how this content is communicated, which is equally critical. While asking the right questions and being eloquent might contribute to a successful collaboration, youth with intellectual disabilities made their decision to participate to a large extent based on how they perceived their potential collaborators (Knox et al. 2000). Our personal characteristics and how we presented ourselves mattered to our co‐researchers.

#### 3.2.4 Theme 4: Ensuring Process Consent

Fourth, the criticality of process consent was observed (Knox et al. 2000). We did this process by seeking consent each day we met for training and for interviews. We asked them daily whether they wanted to continue with the process or not, and if they had any concerns they needed us to address or support them with. After that, we would begin by reflecting on the previous day, then have a discussion around what we all hoped to achieve as a team for the day. The co‐researchers would ask questions and talk about how they felt about the day. It was important to keep explaining and being open to answering as many questions as were repeatedly asked to ensure process consent and full participation. In these ways, we addressed issues of empowerment, social justice and rights (Wickenden 2024).

### 3.3 Step 4. Planning

Finally, we consider the key contributions of this inclusive pilot project, guided by prompts such as how these reflections may inform future approaches to support more inclusive and contextually responsive co‐research practices.

#### 3.3.1 Key Contributions of This Inclusive Pilot Study Implementation

Currently, there is increasing evidence of the inclusion of children and youth as partners in research in Africa (Bailey et al. 2014; Kahonde 2023), as researchers identify the relevance of such inclusive strategies (Sikapa et al. 2024; Wickenden 2024). Although still very limited, various inclusive research emanating from Africa supports the argument that young people can be valuable collaborators in research, bringing in unique perspectives on the processes and implications of research implementation (Bailey et al. 2014; Sikapa et al. 2024). Wanjagua et al. (2024) implemented inclusive research in Kenya and warned of the recurring dangers of the exploitation of persons with intellectual disabilities when there are no supporting structures intentionally set up to include them within society. However, even when there is inclusion, it occurs more within the social sciences, while the health sciences are further behind in this area. In designing and implementing this research from the health sciences perspective, this paper contributes to inclusive research discourse within the health sciences. In practising inclusive research within this space, we were intentional in ensuring that youth with disabilities are seen and respected and that their opinions count towards this process.

A critical aspect of inclusive research is the shared learning from individual contributions that occur and a co‐construction of knowledge within the research process. Disabled persons' active involvement in research is now being seen as essential (Wickenden 2024). This essentialism was foregrounded in this study as the co‐researchers made the university researchers aware of fundamental changes required in the data collection approach (Woelders et al. 2015). Their presence also made the interview process less overwhelming for their peer participants.

Inclusive research has the potential to challenge stigma and stereotypes about persons with intellectual disabilities as non‐productive citizens. Conducting inclusive research showcased the capacity of these youth with intellectual disabilities to gain an income and grow their confidence and respect because they received some payment for the research work they did. These benefits contribute to legitimising and valuing the authenticity and capacities of youth with intellectual disabilities so that people in their communities may perceive them differently than before. The fact that the co‐researchers earned some stipend is indeed novel to their families and community in our context, as they are often perceived as incapable and totally dependent on others.

While most of the concepts related to inclusive research above speak to egalitarianism and the greater good of ensuring voice through inclusion, youth with intellectual disability still exist within the spectrum of either being accepted as part of the community or suffering prejudice in many communities globally (Bevan‐Brown 2013). Mkabile and Swartz (2020) state that stigma and safety remain concerns for persons with intellectual disabilities and their families within the South African context. We contend that one way to foster sustainable inclusion is through more collaborative community‐based engagements with persons with intellectual disabilities. Inclusive research is needed to facilitate and contribute to this call for transformative social change at a personal, collective and communal level.

Additionally, researchers who have been involved in inclusive research are encouraged to share their learned knowledge, best practices and recommendations (Kahonde 2023; Woelders et al. 2015; Wanjagua et al. 2024). The reflections in this paper have reiterated the relevance of inclusive research within the South African and African context because inclusion is a right (Robinson et al. 2022; UN 2006).

More inclusive research is needed to impact both co‐researchers with intellectual disabilities and the university researchers. We advocate for a guided or scaffolded approach to facilitate inclusion. Building communities of practice in inclusive research could support novice researchers in academia and beyond to sufficiently prepare to conduct inclusive research. In this way, the predominantly deficit and disempowering view of society and marginalisation of youth with intellectual disabilities, especially within inequitable contexts, may begin to be addressed.

It is necessary for university researchers to learn how to hold the space while respectfully allowing the co‐researchers to co‐lead. The process of repeating and explaining challenged us to become quite adept at simplifying complex concepts (Woelders et al. 2015). This simplification was helpful in bringing even more clarity to us in the process. Importantly, this type of research should be truly meaningful and collaborative and not only a tick box or checklist. Reflexivity within inclusive research is important. This reflexivity and co‐leading are particularly important to address the power sharing between the two groups as the collaboration unfolds.

## 4 Limitations

We acknowledge the limited scope of involvement of the co‐researchers, as they were only involved in data gathering and not data analysis. The co‐researchers were also initially not part of formulating the research questions and the first draft of the interview questions; they were trained for the co‐implementation. This limitation meant that some adjustments had to be made to the interview questions during the workshop stage when they were drawn in to partner. Going forward, planning for co‐researchers needs to occur when conceptualising the research, not as an ‘add‐on’. A single location was used for this pilot study, as the researchers felt that using a single location is adequate for a pilot study. Another limitation is that sociodemographic factors were not a consideration during the selection of the location for the pilot study, as we could only work in an area where there was active youth participation in the SOI Healthy Communities programme.

## 5 Conclusion

The CDRA Action Learning Cycle (CDRA 2005) offers a deliberate process for engaging in inclusive research by working through action (experience), reflection, learning and planning. Its cyclical orientation enables a more reflexive and responsive approach to co‐research, opening space to interrogate assumptions, attend to participation and incrementally strengthen inclusive practice. Our reflections confirm that co‐researchers could be involved in the entire research process. The co‐researchers need to be a part of question formulation and the design of the study so that they are fully and authentically part of every decision made, helping to shape and modify the research right through to the publication phase as well as other forms of accessible research dissemination (Wickenden 2024). Such an approach would confront the longstanding critique of inclusive research, where some roles are assigned to co‐researchers with disabilities, and other roles are retained for researchers who are in academia (Nind 2011). Inclusive research should strive to remove any distance between university researchers and persons with intellectual disabilities, and support shared decision‐making (Fletcher‐Watson et al. 2021). This requires some humility and courage on behalf of academic researchers to challenge their assumptions and biases around inclusive research, as well as to embrace a different approach to research. This paper seeks to demonstrate that inclusive research is both a moral and practical engagement (Fletcher‐Watson et al. 2021) that enriches and changes both participants, co‐researchers and researchers as it emancipates all.

## Author Contributions

All authors contributed to the conceptualisation and writing of this paper.

## Funding

This paper is connected to a broader evaluation study which was made possible through a Special Olympics International grant for the Healthy Communities qualitative evaluation. This assistance is gratefully acknowledged.

## Consent

Consent was sought and received from the co‐researchers who are part of this pilot study and their parents.

## Conflicts of Interest

The authors declare no conflicts of interest.

## Data Availability

The data that support the reflective findings of the methodological processes of implementing this study are available from the corresponding author upon reasonable request.
